# IdentiCS – Identification of coding sequence and *in silico *reconstruction of the metabolic network directly from unannotated low-coverage bacterial genome sequence

**DOI:** 10.1186/1471-2105-5-112

**Published:** 2004-08-16

**Authors:** Jibin Sun, An-Ping Zeng

**Affiliations:** 1 Department of Genome Analysis, GBF-German Research Center for Biotechnology, Mascheroder Weg 1, Braunschweig, 38124, Germany

**Keywords:** low-coverage, unfinished, genome sequence, annotation, coding sequence, *in silico *reconstruction, visualization, comparison, metabolic network, *Salmonella typhimurium*, *Klebsiella pneumoniae*

## Abstract

**Background:**

A necessary step for a genome level analysis of the cellular metabolism is the *in silico *reconstruction of the metabolic network from genome sequences. The available methods are mainly based on the annotation of genome sequences including two successive steps, the prediction of coding sequences (CDS) and their function assignment. The annotation process takes time. The available methods often encounter difficulties when dealing with unfinished error-containing genomic sequence.

**Results:**

In this work a fast method is proposed to use unannotated genome sequence for predicting CDSs and for an *in silico *reconstruction of metabolic networks. Instead of using predicted genes or CDSs to query public databases, entries from public DNA or protein databases are used as queries to search a local database of the unannotated genome sequence to predict CDSs. Functions are assigned to the predicted CDSs simultaneously. The well-annotated genome of *Salmonella typhimurium *LT2 is used as an example to demonstrate the applicability of the method. 97.7% of the CDSs in the original annotation are correctly identified. The use of SWISS-PROT-TrEMBL databases resulted in an identification of 98.9% of CDSs that have EC-numbers in the published annotation. Furthermore, two versions of sequences of the bacterium *Klebsiella pneumoniae *with different genome coverage (3.9 and 7.9 fold, respectively) are examined. The results suggest that a 3.9-fold coverage of the bacterial genome could be sufficiently used for the *in silico *reconstruction of the metabolic network. Compared to other gene finding methods such as CRITICA our method is more suitable for exploiting sequences of low genome coverage. Based on the new method, a program called IdentiCS (Identification of Coding Sequences from Unfinished Genome Sequences) is delivered that combines the identification of CDSs with the reconstruction, comparison and visualization of metabolic networks (free to download at ).

**Conclusions:**

The reversed querying process and the program IdentiCS allow a fast and adequate prediction protein coding sequences and reconstruction of the potential metabolic network from low coverage genome sequences of bacteria. The new method can accelerate the use of genomic data for studying cellular metabolism.

## Background

Knowledge about the metabolic network of an organism is essential for understanding its physiology and phenotypic behavior. A comprehensive understanding of the metabolic network at the system level is particularly important for both biotechnological and biomedical research and is now made possible by rapid advances in genome sequencing and functional genomics. *In silico *reconstruction of metabolic networks from genome sequences of organisms represents a starting point for a systematic analysis of metabolism [1-3]. The functionality of the potential metabolic network of a given organism can then be further experimentally studied by system perturbations at both physiological and genetic levels [4].

**Figure 1:**
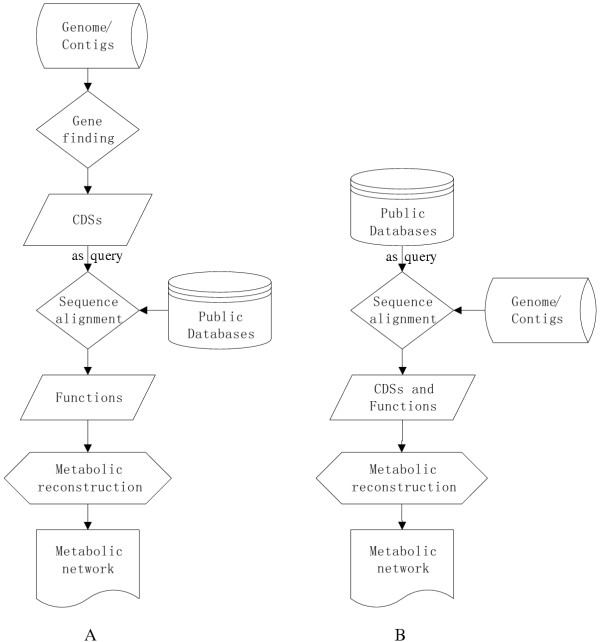
Schematic illustration of methods for reconstructing the metabolic network from genome data: (A) conventional three-step method based on coding sequence (CDS) prediction, function assignment and metabolic reconstruction; (B) proposed two-step method based on a simultaneous and direct identification of coding sequences and their functions from raw genome sequences. Note that the query process has been reversed in the new method in comparison to the conventional method.

Several methods and tools have been recently developed for the reconstruction, visualization and analysis of metabolic networks. These include general static metabolic network tools such as the Kyoto Encyclopedia of Genes and Genomes (KEGG) [5], the Boehringer Mannheim metabolic charts [6,7] and dynamic or potentially dynamic tools such as WIT [3], MPW [8], EcoCyc [9,10] and PathFinder [11]. Metabolic network reconstruction is generally based on the identification of metabolic enzymes and the corresponding biochemical reactions in a specific organism. For this purpose the EC numbers of all possible enzymes need to be determined. The set of EC numbers of an organism may be obtained from the genome annotation. This conventional approach of metabolic network reconstruction is briefly summarized in Fig. 1A. It covers three successive steps: (1) gene finding, (2) database searching and function assignment and (3) metabolic reconstruction. In the first step, genes or coding sequences (CDSs) are predicted from the genome data using programs such as Glimmer [12], GeneMarkS [13], ZCURVE [14] or CRITICA [15]. Then, coding sequences are used as queries and compared to sequence databases such as GenBank, GenPept and SWISS-PROT or to databases of protein domains and functional sites such as InterPro [16], PROSITE [17], Pfam [18] etc. Based on the similarity, the function of the database entry may be assigned to a CDS as its annotation. From these function assignments the metabolic network can be constructed. This three-step approach is used for example in the WIT system [3]. Efforts were also made in some bioinformatic systems such as WIT to reconstruct metabolic networks from incomplete genome data [3]. The program suite ERGO™, a commercial version of WIT, integrates over 400 finished and unfinished genomes into a comprehensive network of metabolic and non-metabolic pathways [19]. No details about the WIT approach have been published and merely some information about 55 annotated genomes (Status: March 2004) is publicly available on the website of WIT [20].

The three-step method starting from gene finding has several drawbacks for the reconstruction of the metabolic network from incomplete or unfinished genome sequences. In unfinished sequences of a genome, especially in sequences with a low genome coverage (e.g. less than 4 fold), there may be many sequencing errors that do not warrant an accurate prediction of genes [21]. For example, the start or stop positions of CDSs may not be accurately predicted. Protein sequences translated from these CDSs may be completely wrong because of coding frame shifts. Fusion CDSs may be predicted, to which the function assignment is difficult. Moreover, a CDS that normally appears as one CDS in other organisms may be predicted as several smaller fragmented CDSs. On the other hand, existing CDSs may not be found at all, either because of sequencing errors or because of limitations of the gene finding software. For eukaryotes, the prediction of CDSs is even more difficult because of the existence of introns.

To avoid these problems, alternative methods are required for directly reconstructing metabolic networks from unfinished genome data. Sequencing and annotation are still time and resource consuming. An as early as possible exploitation of the genome data is of importance for functional genome research. In this work, we propose a method to identify coding sequences for proteins (particularly for metabolic enzymes) directly from unannotated low-coverage genomic data for *in silico *reconstruction of the metabolic network. The method is demonstrated with genome data from two organisms. A program combining automatic prediction and function assignment of CDSs with a visualization and comparison of metabolic networks of different organisms is also delivered.

## Principle of the new method

The principle of the new method is schematically shown in Fig. 1B. In comparison to the conventional three-step method (Fig. 1A) our method can be called a two-step approach. To avoid the separate step of gene finding in the conventional methods, we propose to reverse the searching relationship between public databases and the query sequence: gene or protein sequences from public databases are taken as queries, while the sequences in the unannotated genome of a given organism are treated as a local database that can be searched using a standalone algorithm of BLAST [22]. This results in the prediction of possible CDSs in the genome and simultaneously their functions. Functional information about these CDSs is then used to reconstruct the metabolic network. Thus, our method can significantly simplify the process of CDS prediction and metabolic network reconstruction. By skipping over the separated steps of gene-finding and function assignment, our method can avoid or relax some of the problems of the traditional methods mentioned above.

## Results and Discussion

### Evaluation of IdentiCS for identifying protein coding sequences from genome sequences of *S. typhimurium *LT2

To examine the applicability of the method proposed, the well-annotated genome sequences of *S. typhimurium *LT2 [23] are used as "standard of truth" for statistic evaluation. According to the annotation given in the KEGG database, *S. typhimurium *LT2 has 4449 CDSs, out of which 1218 have been annotated with enzyme EC numbers. These CDSs encompass 656 different unique EC numbers. The reliability of CDS prediction by the program IdentiCS is evaluated by using a nucleotide database (KEGG genome) and a combined protein database (SWISS-PROT + TrEMBL + TrEMBL update) separately. The annotated coding sequences or proteins of *S. typhimurium *were filtered out of these databases before sequence alignment. All CDSs having an E-value less than 10^-10 ^were accepted and submitted for comparison with the KEGG annotation of *S. typhimurium*. The results are summarized in Table 1. 92.6% and 97.7% (sensitivity) of the CDSs in the original annotation of *S. typhimurium *are identified by using the KEGG genome database and the whole protein database SWISS-PROT and TrEMBL, respectively. The sensitivity on the nucleotide level (91.1% and 98.2% for the two databases respectively) is similar as on the CDS level. These results suggest that the SWISS-PROT-TrEMBL based approach is more preferable than the KEGG genome based approach for our method. It is understood that the combined protein database SWISS-PROT and TrEMBL contains almost all of the known protein sequences available in public databases (including proteins *in silico *translated from nucleotide sequences) while the KEGG database contains only a limited number of sequenced and annotated genomes. The difference becomes more significant if the organism studied is evolutionarily far from any organism whose genome is completely sequenced and annotated.

The specificity of the method is about 81–82% on the CDS level and 87.2–94.9% on the nucleotide level for the KEGG genome database and the whole protein database SWISS-PROT and TrEMBL. The moderate specificity on CDS level is due to the relatively high amount of additionally predicted CDSs (false positive). It should be mentioned that all the additionally predicted CDSs have quite strong statistic significance (most of them with an E-value 1E-20 – 1E-40). These additional CDSs may be missed in the original annotation and could in fact represent good candidates for an improved annotation of the genome.

The inconsistence rate by IdentiCS is as low as 0.35% for the KEGG genome database and 0.64% for the SWISS-PROT and TrEMBL protein database, indicating the reliability of our method.

**Figure 2:**
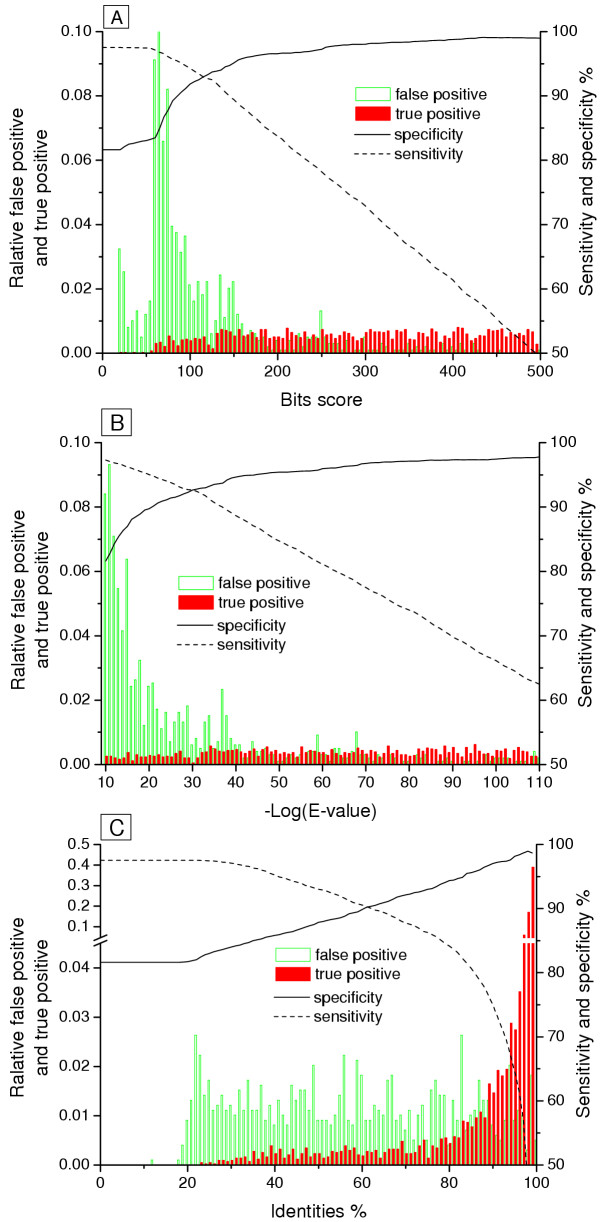
Distribution of false positive, true positive, sensitivity and specificity as functions of bits score (Fig. 2A), E-value (Fig. 2B) and identity (Fig. 2C). The bars for the distribution of false positive and true positive indicate the corresponding number of CDSs in a given interval of the statistic parameter (5 for bits score, 1 for -log(E-value) and 0.01 for identity) divided by the number of all false positive or true positive CDSs submitted for analysis. The lines show the specificity and sensitivity change along the cut-off scoring values.

In the above mentioned evaluation of the method, the cut-off E-value is less than 1E-10 for the CDS prediction. Further the effects of different scoring parameters (i.e. bits score, E-value and identities) and their cut-off values on the CDS prediction by using the database SWISS-PROT and TrEMBL are examined. (Fig. 2A,2B,2C) show the distribution of the true positive CDSs, false positive CDSs, sensitivity and specificity as function of bits score, E-value and identities respectively. The major part of the false positive CDSs is found in the regions of bits score less than ca. 80 and E-value larger than 1E-15. The specificity increases with the bits score and E-value in a form of a saturation curve while the sensitivity decreases almost linearly. This indicates that the prediction performance can be further optimized by choosing appropriate cut-off parameters. If the CDSs with bits score less than 75 (their corresponding E-values are higher than 1E-15 in most cases) are rejected, the false positive will decrease by 40% (from 987 to 602) while the false negative will increase from 110 to 144 (Table 2). The prediction specificity increases from 81.5% to 87.7% at the expense of a slight decrease in the sensitivity from 97.5% to 96.8%. The specificity of IdentiCS can be further improved by combining a third criterion, i.e. Identities > = 25% (Table 2). Thus, the sensitivity and specificity of IdentiCS for CDS prediction are satisfactory and can be balanced to certain extent by choosing proper scoring parameters.

### Identification of enzyme-coding sequences in *S. typhimurium *LT2

For the reconstruction of metabolic network it is desired to know the enzyme-coding sequences, and especially the EC-number containing enzymes in an organism. The possibility to use the EC number-containing subset of the combined protein database SWISS-PROT + TrEMBL to identify enzyme-coding sequences in *S. typhimurium *LT2 and thus to further reduce the computation time for constructing the metabolic network is examined (Table 3). 1894 of EC-number containing CDSs (EC-CDSs) are identified. Of the 1218 originally annotated EC-CDSs, 98.9% of them are identified with an annotation inconsistence rate of as low as 0.08%. The specificity appears to be relatively low due to the large number of false positives. However, if the prediction of EC-CDSs is compared to all the originally annotated CDSs, the number of true positive EC-CDSs is increased from 1204 to 1813 and the number of false positive EC-CDSs is decreased from 690 to 55, resulting in a specificity of 97.1%. The inconsistence rate still remains at a low level, indicating the prediction and function assignment for the additionally predicted EC-CDSs is correct and their EC numbers are missed in the original annotation. This can helps in reconstructing a more complete metabolic network.

The KEGG genomes based prediction is also evaluated for its ability to predict the enzyme-coding sequences. 95.4% of the CDSs originally annotated to have an EC-number are correctly predicted and assigned with EC numbers. This value is slightly lower than the one based on the SWISS-PROT+TrEMBL database. The more complete protein databases are therefore more suitable for EC-CDS identification as well.

### Identification of enzyme coding sequences with different coverage of genome sequences of *K. pneumoniae*

Both the KEGG genomes and SWISS-PROT-TrEMBL databases are used to identify enzyme-coding sequences from the 3.9-fold and 7.9-fold coverage genome sequences of *K. pneumoniae*. From the 3.9-fold coverage genome, IdentiCS identified 1169 and 1342 EC-CDSs by applying the KEGG genome database and SWISS-PROT-TrEMBL databases, respectively, whereas from the 7.9-fold genome sequences 1158 and 1495 EC-CDSs, respectively. As in the case of *S. typhimurium, *IdentiCS identified 15% to 30% more EC-CDSs with queries from SWISS-PROT-TrEMBL than with queries from KEGG for the two versions of *K. pneumoniae *genome sequences respectively. The number of EC-CDSs identified for *K. pneumoniae *is comparable to that identified for *S. typhimurium *with the respective databases. They are also comparable to the number (1156) of annotated EC-CDSs of *E. coli *based on the KEGG genome database. With the method proposed by Ma and Zeng [24], the structure and evolution distance of the metabolic networks of these three organisms and other 47 bacteria are compared. The metabolic network of *K. pneumoniae *is found to be most similar to those of *E. coli *and *S. typhimurium *(data not shown). Thus, the predicted number of enzyme-encoding sequences for *K. pneumoniae *appears to be reasonable. With the same 3.9-fold coverage genome sequences of *K. pneumoniae*, the method of WIT predicted 2650 EC-CDSs which are twice the number of EC-CDSs in *E. coli *and *S. typhimurium*. The EC-CDSs predicted by WIT are significantly smaller and fragmented, possibly because of the presence of too many errors in the unfinished genome sequences. The fragmentation problem was overcome in our method that leads to a significant reduction in the number of identified EC-CDSs. The less false positive EC-CDSs will further simplify experimental design such as for microarray to examine the metabolic network.

A comparison of the unique EC numbers of EC-CDSs identified from the two different versions of genome sequences and by the different approaches reveals that the results of these different combinations share over 80% of common EC numbers (Table 4). The WIT version contains more EC numbers than other versions, obviously because the criteria used in our approaches allow a region to have only one function or an EC number whereas the method used by WIT allows more. With the 3.9-fold genome sequences both KEGG and SWISS-PROT based methods identified a certain number of EC numbers (44 and 81, respectively) that are not identified by WIT. Interestingly, the two different versions of genome sequences result in very close EC numbers. The KEGG based approach identified only 11 (1.68%) more and the SWISS-PROT-TrEMBL based approach identified merely 57 (7.76%) more by using the 7.9-fold genome sequences than by using the 3.9-fold genome sequences (Table 4). This indicates that the 3.9-fold coverage genome sequences result in a fairly good estimation of enzyme-coding sequences for the purpose of an *in silico *reconstruction of the metabolic network of *K. pneumoniae*. It would be of interest to examine if this also applies to other organisms or even lower genome coverage. The use of lower genome coverage sequences for studying cellular metabolism will greatly accelerate the exploitation of genome sequencing projects.

The EC numbers of *K. pneumoniae *as identified by the combination of 7.9-fold genome sequences and SWISS-PROT and TrEMBL databases are summarized in Table 5 in terms of different functional categories. More than half of the enzymes are involved in the metabolism of carbohydrates, amino acids, cofactors and vitamins. 22.2% – 47.6% of the enzymes in the different KEGG metabolism categories can be found in *K. pneumoniae *(Table 5). These values are comparable to the values of several evolutionarily closely related strains such as *Escherichia coli, S. typhimurium*, *S. typhi*, *Pseudomonas aeruginosa *and *Yersinia pestis*.

### Comparison of IdentiCS and CRITICA for identifying coding sequence from low coverage genome sequences

CRITICA is a well-developed program for the prediction of coding sequences [15]. It combines the comparative analysis of DNA sequences with noncomparative methods (i.e. dicodon bias). We compared CRITICA and IdentiCS for predicting CDSs from the two different versions of *K. pneumoniae *genomic sequences. The comparison is done on the basis of all the CDSs including non-enzyme coding sequences. The results are summarized in Table 6.

From the 3.9-fold coverage genome data, CRITICA predicts 6734 CDSs with a cut-off p-value = -4 suggested by Badger and Olsen [15], while IdentiCS predicted 5650 CDSs (with a cut-off E-value = 1E-10). 94.0% of the CDSs predicted by CRITICA are covered by the prediction of IdentiCS. O, In many cases two or more smaller CDSs predicted by CRITICA are covered by a CDS predicted by IdentiCS, obviously because of the relatively high sequencing errors in the 3.9-fold coverage genome data. CRITICA predicts 29 fusion coding sequences. Since they have similarities to two different functions, function assignment to this kind of fusion CDSs is uncertain. Half of the CRITICA-specific CDSs have p-values between 1E-4 and 1E-10. In comparison, of the 1348 CDSs merely predicted by IdentiCS, all have E-values less than 1E-10, 27% have E-values less than 1E-40; all the CDSs have an identity greater than 20% and 60% have an identity greater than 50%, indicating that the predictions by IdentiCS have a high confidence.

From the 7.9-fold coverage genome data, CRITICA predicts 5135 CDSs. This number is much less than the CDS number predicted from the 3.9-fold coverage. This may be explained by the significant decrease of sequence errors in the 7.9-fold genome data. In contrast, CDSs predicted by IdentiCS are only 389 less than that predicted from the 3.9-fold genome data. 93.9% of the CRITICA predictions are covered by the 4512 CDSs predicted by IdentiCS. Only 8 CDSs predicted by CRITICA span two or more CDSs. This shows that the increase of sequence quality increases the precision of the prediction of CRITICA. Again, the IdentiCS-specific predictions have a high confidence: all with E-values less than 1E-10 and amino acid sequence identities greater than 20%, more than 50% with E-values less than 1E-20 and identities greater than 50%. The fact that in some cases fusion CDSs are predicted by CRITICA and in other cases many highly potential coding regions are not predicted as CDSs indicates a shortcoming in this algorithm for low quality contigs. When CRITICA finds a coding region with a high score, it tries to find the start and stop codons by extending this region to both upstream and downstream with the conditions of not decreasing the total score after extension. Sequencing errors, especially translation shifts, make it difficult for CRITICA to calculate the extension score correctly. In such cases, the algorithm used by IdentiCS does not need to locate the start and stop codons. Transcription frame shifts also have less interference to IdentiCS because it does not use predicted coding sequence as queries but uses entries from public database to search for coding sequences in the raw genome sequences of an organism. These features make IdentiCS more suitable for identifying possible protein-coding regions from low-coverage error-containing raw genome sequences than other available approaches.

### Reconstruction and visualization of metabolic networks for comparison

With the identified enzyme-encoding sequences discussed above the potential metabolic networks of *S. typhimurium *and *K. pneumoniae *can be reconstructed and compared to other organisms. The reconstruction of metabolic networks can be done in a similar way as based on CDSs from annotated genome sequences as recently described by Ma and Zeng [1]. Briefly, from the identified EC numbers of CDSs, the set of biochemical reactions involved in the organism can be established with the help of a reaction database (i.e. a revised version of LIGAND [5] or BRENDA [25]). From the reaction set, a connection matrix is obtained that can be used to represent the metabolic network as a directed graph for computational analysis.

**Figure 3:**
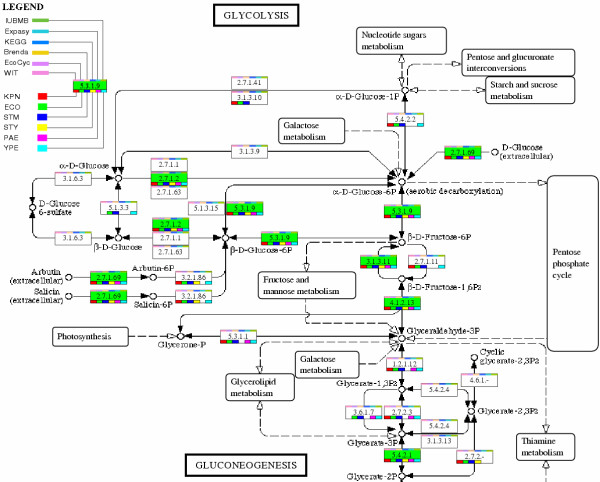
Glycolysis pathways as example for demonstration of metabolic comparison among different organisms. KPN: *K. pneumoniae *MGH78578, ECO: *Escherichia coli *K-12 MG1655, STM: *Salmonella typhimurium *LT2, STY: *Salmonella typhi*, PAE: *Pseudomonas aeruginosa *PA01, YPE: *Yersinia pestis *strain CO92. Green background with enzyme EC number means that this enzyme exists in all the compared organisms. Colored rectangles under the EC number box represent links to the strain-specific annotation of the corresponding enzyme. Colored bars above the EC number link the enzyme to the corresponding entry in different public databases.

For a straightforward visualization of the biochemical reactions related to a specific organism and especially for comparing the metabolisms of different organisms, the KEGG metabolic maps act as a blueprint for visualization of metabolic networks in this work. Reconstruction, comparison and visualization of the metabolic network have been integrated in the program IdentiCS as a built-in component. It can work directly with the coding sequences and their functions predicted by IdentiCS or with existing annotation files such as the those in Microsoft Excel format or in GenBank flat file format. To use the KEGG maps for metabolic reconstruction, comparison and visualization information from the KEGG metabolic pathways is reformed into an Excel template. Metabolic network reconstruction is realized by mapping the identified enzymes (EC numbers) to the KEGG metabolic maps. Different similarity levels of the identified enzymes can be displayed in different colors. All identified enzymes in the map are marked and linked to their annotations. Web links to other Internet databases such as IUBMB [26], BRENDA [25], WIT [3], KEGG [5], Ecocyc [9,10] and SWISS-PROT [27] are also integrated to offer the user a fast tool to access the relevant information. For metabolic network comparison, strain-specific colored boxes are drawn on the lower part of the EC number rectangle if that strain possesses this enzyme as demonstrated in Fig. 3 for the glycolysis pathways. This box is linked to the original annotation of the enzyme in that strain. The background of the enzyme box becomes green if this enzyme is found in all the compared organisms. In such a way an informative metabolic network is generated which can serve as a starting point for functional and comparative analysis of the metabolism of organisms under study.

## Conclusions

The use of genome sequences from *S. typhimurium *and *K. pneumoniae *demonstrated the applicability and reliability of the new method proposed for *in silico *identification of protein coding sequences from unannotated genome sequences. The use of protein sequence databases SWISS-PROT and TrEMBL is more favorable than the use of KEGG genome database for identifying coding sequences and thus for metabolic network reconstruction. Furthermore, the method allows an adequate reconstruction of the potential metabolic network from sequence data with low coverage (e.g. < 4 fold) of the bacterial genome as shown for *K. pneumoniae*. Together with the algorithms for the automatic annotation of sequences, the visualization and comparison of metabolic networks, the method and program developed in this work can accelerate the use of genomic data for studying cellular metabolism.

## Methods

### Database preparation

The applicability of the method proposed above was examined with the genome sequences of two organisms, namely *Salmonella typhimurium *LT2 and *Klebsiella pneumoniae*. The genome of *S. typhimurium *LT2 has been completely sequenced and well annotated [23]. Thus, the annotated genome sequences of *S. typhimurium *LT2 serve as a reference to evaluate the accuracy of the proposed method. The sequences and annotation for *S. typhimurium *LT2 were downloaded from KEGG [28](version of Dec. 18. 2003). The genome of *K. pneumoniae *has been recently sequenced and the annotation is still in progress. Two different versions of the raw genome data of *K. pneumoniae *(3.9-fold whole genome shotgun coverage in 920 contigs and 7.9-fold coverage in 341 contigs) obtained from the Genome Sequencing Center of Washington University [29] were examined in this study. Each version of the raw genome data was formatted as a local database for BLAST [22].

Two types of databases are used in this work for the prediction and function assignment of CDSs for a given organism, namely the nucleic acid database from KEGG and the non-redundant protein sequence databases from SWISS-PROT, TrEMBL and TrEMBL updates. The reason to choose the genome database from KEGG as query but not from other nucleic acid databases such as GenBank or EMBL is that KEGG contains the most extensive EC numbers for enzymes that are needed for reconstructing metabolic networks. Therefore, the genome database of KEGG version can serve as an EC number source and be used for the purpose of comparative analysis of genome-based metabolism. In contrast, the flat data files from GenBank and EMBL do not contain the necessary enzyme index information in many cases. SWISS-PROT is human-curated and therefore more preferred. SWISS-PROT and its sister database TrEMBL (SWISS-PROT Release 42.7, TrEMBL Release 25.7, released on 15 Dec. 2003) were obtained from the Swiss Institute of Bioinformatics [30]. Not "fasta" format files but SWISS-PROT flat files were used because the enzyme EC numbers may not be included in the fasta format files available on the FTP site. Entries in the databases that do not contain EC numbers can be filtered out before the sequence alignment step to shorten the computational time if the purpose is merely to identify metabolic enzymes and to reconstruct the metabolic network. For identifying all possible CDSs, the complete SWISS-PROT and TrEMBL databases are used.

### Automatic prediction and annotation of protein-coding sequences

The annotation process is based on similarity comparison as normally used in other annotation processes. The difference is that in our approach the gene or protein sequences from public databases are used as queries to search and locate similar ones in the raw genome sequences. When proteins from public database are used as queries, the tblastn algorithm in the BLAST program is applied that compares the query to all six translation frames of the unannotated DNA sequences. The dynamic translation of a small genomic database takes much less system resource than the translation of a large public database as in the conventional methods. Our method can thus be realized on a common PC system, especially when merely a subset of the public database is considered, for example for the purpose of identifying metabolic enzymes for metabolic network reconstruction.

Because of sequence errors (especially the translation shift and abnormal stop codon) and the local alignment nature of the BLAST algorithm, the BLAST research may report several small alignments between different parts of the query protein or gene and different parts of a genomic contig even if there should be only one alignment in the reality. In this situation, the tfasty34 program in the FASTA3 suite [31] should give a better alignment since the translation shift is considered. But the tfasty34 program runs very slowly in our test and is therefore not used here for large-scale genomic alignment. In this work, fragment(s) of a genomic contig are joined to the genomic fragment that has the highest alignment score, resulting in a larger CDS fragment if:

1. they are coded on the same strand of the same genomic contig as the highest score fragment. In other words, all of these fragments must be translated either in positive or in negative frames.

2. the alignments have an identity level not lower than 80% of the identity level of the highest score alignment.

3. the generated larger sequence region has alignment gaps or extensions not more than 20% of its length.

Since many queries can be similar to the same region on a genomic contig and sometimes they may have different function annotations, the program must judge and choose one annotation for this region. The decision is made by applying the following criteria:

1. Each region normally has only one function. Here the region represents a piece of nucleotides either on the positive strand or on the negative strand of a DNA molecule. The same physical position on different strands of a DNA molecule can belong to different regions, and can therefore have a different function assignment. Although there are examples in some viruses that a region can code different proteins depending on the transcription frame, it happens very rarely in other organisms. The user can assign a tolerance value (e.g. 60 bp) to allow two successive regions to overlap each other to some extent.

2. Highest similarity principle. If a query gene or protein has a similarity to a CDS higher than other queries, then the function of this query gene or protein is assigned as the annotation of the CDS. Bits score is used as a measure for similarity first. If two queries have the same bits score, then the identity level in percentage is taken as a second measure for similarity. If both bits score and identity are the same and these two entries have different function annotation (rarely occurred) then both of their functions are assigned to that region.

3. Closest evolutionary relationship. If two or more query genes or proteins are comparably similar (e.g. the difference between their identity levels is lower than 5%) to a CDS but have different function, the evolutionary relationship between these organisms is further considered. The annotation of the organism that is mostly related to the studied organism from the viewpoint of metabolic evolution is transferred to the unknown CDS. The evolutionary relationship between different organisms and the one studied is established with the method of Ma and Zeng [24] after the initial function assignment for the CDSs with the highest similarity criteria.

In this way, the coding sequences of a genome are identified and annotated at the same time. No second large-scale sequence alignment is needed. Once all the software and databases are prepared, our program which is called IdentiCS (Identification of Coding Sequences from Raw Genome Sequences) can reconstruct the metabolic network of an organism with about 5 million base pairs of raw genome data. The computing time is less than 8 hours on a PC with 2.8 GHz Pentium 4 CPU and 512 MB memory. This program works together with Microsoft Excel under Windows environment.

### Statistic evaluation

For a more detailed examination of our method, the results are evaluated separately for the prediction of CDSs and their function assignment, although our method integrates these two aspects into one step. The terms true positive (TP), false negative (FN) and false positive (FP) are used to calculate the sensitivity and specificity of CDS prediction in comparison with CDSs in the original annotation. The terms "sensitivity" and "specificity" are defined according to Burset and Guigo [32]:


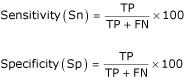


We also evaluated the terms TP, FN and FP on nucleotide level according to Burset and Guigo [32] and calculated the corresponding sensitivity and specificity as above. It should be mentioned that a true positive CDS does not necessarily mean that its function assignment is also correct. The terms consistence and inconsistence are used to describe whether a true positive CDS has the same function assignment as in the original annotation or not. Correspondingly, an "inconsistence rate" is used and defined as:


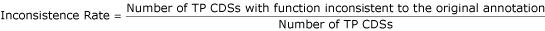


## Authors' contributions

JS is the developer of the method and the program. APZ supervised the study. Both authors read and approved the final manuscript.

## Figures and Tables

**Table 1:** Evaluation of the method IdentiCS for identification of CDSs in the genome of *Salmonella typhimurium*. KEGG: KEGG genome database; SW: SWISS-PROT + TrEMBL + TrEMBL updates.

Database	KEGG	SW
True positive	4121	4339
False positive	907	987
False negative	328	110
Sensitivity %*	92.6 (91.1)	97.7 (98.2)
Specificity %*	82.0 (94.9)	81.5 (87.2)
Inconsistence rate (%) in TP	0.35	0.64

*: Values shown in parentheses are calculated based on the nucleotide level according to Burset and Guigo[32]

**Table 2:** Effects of different scoring criteria on CDS identification in the genome of *S. typhimurium *using IdentiCS and the database SWISS-PROT and TrEMBL. Criteria 1: E-value < = E-10; Criteria 2: E-value < = E-10 and Bits score > = 75; Criteria 3: E-value < = E-10, Bits score > = 75 and Identities > = 25%

	Criteria 1	Criteria 2	Criteria 3
True positive	4339	4305	4303
False positive	987	602	555
False negative	110	144	146
Sensitivity %	97.5	96.8	96.7
Specificity %	81.5	87.7	88.6

**Table 3:** Evaluation of the performance of IdentiCS for the prediction of EC number -containing CDSs (EC-CDSs) with the EC-number containing subset of the protein database SWISS-PROT and TrEMBL.

	Compared to originally annotated EC-CDSs	Compared to all originally annotated CDSs
EC-CDSs predicted	1894	1894
True positive	1204	1813
False positive	690	55
False negative	14	NA
Sensitivity	98.9%	NA
Specificity	63.6%	97.1%
Inconsistence rate in T.P.	0.08%	3.32%

NA: not applicable.

**Table 4:** Comparison of EC numbers identified with different methods and different versions of the genome sequence of *Klebsiella pneumoniae*. WIT: WIT version of annotation by gene prediction from the 3.9-fold genome sequences; KEGG3.9 and KEGG7.9: annotations of the 3.9-fold and 7.9-fold genome sequences by applying the KEGG genome database. SW3.9 and SW7.9: annotations of the 3.9-fold and 7.9-fold genome sequences by applying SWISS-PROT and TrEMBL protein databases.

Annotation version	Number of unique ECs identified	Version-specific EC numbers* compared to:				
		
		WIT	KEGG3.9	KEGG7.9	SW3.9	SW7.9
WIT	764	-	162	159	152	131
KEGG3.9	646	44	-	4	63	56
KEGG7.9	653	48	11	-	68	57
SW3.9	693	81	110	108	-	15
SW7.9	735	102	145	139	57	-

*The version-X-specific EC number compared to the version Y is referred to EC numbers that are only found in the annotation Version X but not in Y. For example, the KEGG3.9 has only 4 version-specific EC numbers compared to KEGG7.9. On the other side, KEGG7.9 has 11 version-specific EC numbers compared to KEGG3.9.

**Table 5:** Distribution of the unique EC numbers of *K. pneumoniae *in different function categories compared to other organisms (see legend of Fig. 3 for name abbreviations of organisms). The EC numbers for *K. pneumoniae *were identified from the unannotated 7.9-fold coverage genome sequences by SWISS-PROT-TrEMBL based IdentiCS. The EC numbers for other organisms are taken from the KEGG genome annotations. The total number of strain-specific ECs is shown in parenthesis under the strain name.

Function Categories^a^	ECs^b^	KPN (735)	ECO (681)	STM (655)	STY (445)	PAO (573)	YPE (585)
Carbohydrate metabolism	427	151	154	147	101	105	123
Energy metabolism	167	60	62	62	46	61	58
Lipid metabolism	126	27	25	25	17	27	17
Nucleotide metabolism	166	83	82	80	45	66	72
Amino Acid metabolism	561	211	189	189	132	205	183
Other Amino Acids	146	49	45	43	26	47	41
Complex Carbohydrates	184	63	58	55	33	43	53
Complex Lipids metabolism	171	46	38	34	24	31	34
Cofactors and Vitamins	225	104	105	107	74	100	96
Sum of unique EC numbers	1899	567	537	529	349	464	476

^a^: Functional categorization according to KEGG; ^b^: unique EC numbers of corresponding metabolism category calculated from KEGG metabolic pathway maps.

**Table 6:** Comparison of coding sequences (CDSs) prediction by IdentiCS and CRITICA from unfinished genome sequences of *K. pneumoniae *with different genome sequence coverage.

	3.9 × genome data		7.9 × genome data	
	
	CRITICA	IdentiCS	CRITICA	IdentiCS
Number of all CDSs	6734	5650	5135	5261
CDSs shared by both programs	6332*	4302	4823	4512
CDSs merely identified by the respective program	402	1348	312	749

*CDSs predicted by IdentiCS can cover more than one smaller CDSs predicted by CRITICA.

## References

[B1] Ma HW, Zeng AP (2003). Reconstruction of metabolic networks from genome data and analysis of their global structure for various organisms. Bioinformatics.

[B2] Ma HW, Zeng AP (2003). The connectivity structure, giant strong component and centrality of metabolic networks. Bioinformatics.

[B3] Overbeek R, Larsen N, Pusch GD, D'Souza M, Selkov E, Kyrpides N, Fonstein M, Maltsev N, Selkov E (2000). WIT: integrated system for high-throughput genome sequence analysis and metabolic reconstruction. Nucleic Acids Res.

[B4] Ideker T, Thorsson V, Ranish JA, Christmas R, Buhler J, Eng JK, Bumgarner R, Goodlett DR, Aebersold R, Hood L (2001). Integrated genomic and proteomic analyses of a systematically perturbed metabolic network. Science.

[B5] Kanehisa M, Goto S (2000). KEGG: kyoto encyclopedia of genes and genomes. Nucleic Acids Res.

[B6] Michal G (1992). Biochemical Pathways.

[B7] Michal G (1999). Biochemical Pathways.

[B8] Selkov E, Grechkin Y, Mikhailova N, Selkov E (1998). MPW: the Metabolic Pathways Database. Nucleic Acids Res.

[B9] Karp PD, Riley M, Saier M, Paulsen IT, Paley SM, Pellegrini-Toole A (2000). The EcoCyc and MetaCyc databases. Nucleic Acids Res.

[B10] Karp PD, Riley M, Saier M, Paulsen IT, Collado-Vides J, Paley SM, Pellegrini-Toole A, Bonavides C, Gama-Castro S (2002). The EcoCyc Database. Nucleic Acids Res.

[B11] Goesmann A, Haubrock M, Meyer F, Kalinowski J, Giegerich R (2002). PathFinder: reconstruction and dynamic visualization of metabolic pathways. Bioinformatics.

[B12] Delcher AL, Harmon D, Kasif S, White O, Salzberg SL (1999). Improved microbial gene identification with GLIMMER. Nucleic Acids Res.

[B13] Besemer J, Lomsadze A, Borodovsky M (2001). GeneMarkS: a self-training method for prediction of gene starts in microbial genomes. Implications for finding sequence motifs in regulatory regions. Nucleic Acids Res.

[B14] Guo FB, Ou HY, Zhang CT (2003). ZCURVE: a new system for recognizing protein-coding genes in bacterial and archaeal genomes. Nucleic Acids Res.

[B15] Badger JH, Olsen GJ (1999). CRITICA: coding region identification tool invoking comparative analysis. Mol Biol Evol.

[B16] Mulder NJ, Apweiler R, Attwood TK, Bairoch A, Barrell D, Bateman A, Binns D, Biswas M, Bradley P, Bork P, Bucher P, Copley RR, Courcelle E, Das U, Durbin R, Falquet L, Fleischmann W, Griffiths-Jones S, Haft D, Harte N, Hulo N, Kahn D, Kanapin A, Krestyaninova M, Lopez R, Letunic I, Lonsdale D, Silventoinen V, Orchard SE, Pagni M, Peyruc D, Ponting CP, Selengut JD, Servant F, Sigrist CJ, Vaughan R, Zdobnov EM (2003). The InterPro Database, 2003 brings increased coverage and new features. Nucleic Acids Res.

[B17] Falquet L, Pagni M, Bucher P, Hulo N, Sigrist CJ, Hofmann K, Bairoch A (2002). The PROSITE database, its status in 2002. Nucleic Acids Res.

[B18] Bateman A, Birney E, Cerruti L, Durbin R, Etwiller L, Eddy SR, Griffiths-Jones S, Howe KL, Marshall M, Sonnhammer EL (2002). The Pfam protein families database. Nucleic Acids Res.

[B19] The Genomic Database at Integrated Genomics, Inc. http://www.integratedgenomics.com/genomic.html.

[B20] The Academic Site of WIT. http://www-wit.mcs.anl.gov/.

[B21] Mount DW (2001). Bioinformatics: Sequence and genome analysis.

[B22] Altschul SF, Madden TL, Schaffer AA, Zhang J, Zhang Z, Miller W, Lipman DJ (1997). Gapped BLAST and PSI-BLAST: a new generation of protein database search programs. Nucleic Acids Res.

[B23] McClelland M, Sanderson KE, Spieth J, Clifton SW, Latreille P, Courtney L, Porwollik S, Ali J, Dante M, Du F, Hou S, Layman D, Leonard S, Nguyen C, Scott K, Holmes A, Grewal N, Mulvaney E, Ryan E, Sun H, Florea L, Miller W, Stoneking T, Nhan M, Waterston R, Wilson RK (2001). Complete genome sequence of Salmonella enterica serovar Typhimurium LT2. Nature.

[B24] Ma HW, Zeng AP (2004). Phylogenetic comparison of metabolic capacities of organisms at genome level. Mol Phylogenet Evol.

[B25] Schomburg I, Chang A, Ebeling C, Gremse M, Heldt C, Huhn G, Schomburg D (2004). BRENDA, the enzyme database: updates and major new developments. Nucleic Acids Res.

[B26] International Union of Biochemistry and Molecular Biology (IUBMB). http://www.iubmb.unibe.ch.

[B27] The ExPASy (Expert Protein Analysis System) proteomics server of the Swiss Institute of Bioinformatics (SIB). http://www.expasy.org.

[B28] The FTP Site of KEGG Genomes. ftp://ftp.genome.ad.jp/pub/kegg/genomes.

[B29] The Genome Sequencing Center at Washington University Medical School. http://genome.wustl.edu.

[B30] The Non-Redundant Protein Sequence Database. ftp://ftp.expasy.org/databases/sp_tr_nrdb.

[B31] Pearson WR, Misener S, Krawetz SA (1999). Flexible similarity searching with the FASTA3 program package. In Bioinformatics Methods and Protocols.

[B32] Burset M, Guigo R (1996). Evaluation of gene structure prediction programs. Genomics.

